# Robust and Facile Automated Radiosynthesis of [^18^F]FSPG on the GE FASTlab

**DOI:** 10.1007/s11307-021-01609-w

**Published:** 2021-05-20

**Authors:** Richard Edwards, Hannah E. Greenwood, Graeme McRobbie, Imtiaz Khan, Timothy H. Witney

**Affiliations:** 1 School of Biomedical Engineering & Imaging Sciences, King’s College London, St. Thomas’ Hospital, London, SE1 7EH UK; 2 Pharmaceutical Diagnostics, Life Sciences, GE Healthcare, Pollards Wood, Nightingales Lane, Chalfont St. Giles, Buckinghamshire, HP8 4SP UK

**Keywords:** [^18^F]FSPG, FASTlab, Automated synthesis, Positron emission tomography, Cancer imaging, SPE purification

## Abstract

**Purpose:**

(S)-4-(3-^18^F-Fluoropropyl)-ʟ-Glutamic Acid ([^18^F]FSPG) is a radiolabeled non-natural amino acid that is used for positron emission tomography (PET) imaging of the glutamate/cystine antiporter, system x_C_^-^, whose expression is upregulated in many cancer types. To increase the clinical adoption of this radiotracer, reliable and facile automated procedures for [^18^F]FSPG production are required. Here, we report a cassette-based method to produce [^18^F]FSPG at high radioactivity concentrations from low amounts of starting activity.

**Procedures:**

An automated synthesis and purification of [^18^F]FSPG was developed using the GE FASTlab. Optimization of the reaction conditions and automated manipulations were performed by measuring the isolated radiochemical yield of [^18^F]FSPG and by assessing radiochemical purity using radio-HPLC. Purification of [^18^F]FSPG was conducted by trapping and washing of the radiotracer on Oasis MCX SPE cartridges, followed by a reverse elution of [^18^F]FSPG in phosphate-buffered saline. Subsequently, the [^18^F]FSPG obtained from the optimized process was used to image an animal model of non-small cell lung cancer.

**Results:**

The optimized protocol produced [^18^F]FSPG in 38.4 ± 2.6 % radiochemical yield and >96 % radiochemical purity with a molar activity of 11.1 ± 7.7 GBq/μmol. Small alterations, including the implementation of a reverse elution and an altered Hypercarb cartridge, led to significant improvements in radiotracer concentration from <10 MBq/ml to >100 MBq/ml. The improved radiotracer concentration allowed for the imaging of up to 20 mice, starting with just 1.5 GBq of [^18^F]Fluoride.

**Conclusions:**

We have developed a robust and facile method for [^18^F]FSPG radiosynthesis in high radiotracer concentration, radiochemical yield, and radiochemical purity. This cassette-based method enabled the production of [^18^F]FSPG at radioactive concentrations sufficient to facilitate large-scale preclinical experiments with a single prep of starting activity. The use of a cassette-based radiosynthesis on an automated synthesis module routinely used for clinical production makes the method amenable to rapid and widespread clinical translation.

**Supplementary Information:**

The online version contains supplementary material available at 10.1007/s11307-021-01609-w.

## Introduction

The clinical use of positron emission tomography (PET) for the detection and staging of cancer using [^18^F]2-fluoro-2-deoxy-D-glucose ([^18^F]FDG) is a powerful example of how the metabolic reprogramming of cancer is visualized by molecular imaging [1]. The overexpression of amino acid transporters in transformed cells is well-recognized as a biomarker that can also be targeted for diagnostic purposes, for example using radiolabeled non-natural amino acids. Such radiotracers offer advantages over [^18^F]FDG when imaging tumors that are located adjacent to healthy tissues with naturally high glycolytic rates (e.g. the brain) or in cases where the cancer does not exhibit aberrant glucose uptake (e.g. prostate cancer) [2–6]. (S)-4-(3-^18^F-Fluoropropyl)-L-Glutamic Acid ([^18^F]FSPG) is a radiolabeled non-natural amino acid used for PET imaging of the amino acid transporter, system x_C_^-^ [7–15]. The elevated expression of system x_C_^-^ in a wide range of cancer types can be targeted by radiotracers specific to this transporter to provide high contrast images of tumors [7, 11, 16–18]. Clinically, [^18^F]FSPG has been evaluated in hepatocellular carcinoma, non-small cell lung cancer, prostate cancer, and intracranial malignancies [9, 15, 18, 19].

Interest in non-invasively imaging system x_C_^-^ has increased as its central role in modulating cellular redox homeostasis has emerged [17, 20–22]. System x_C_^-^ transports extracellular cystine into the cell in exchange for intracellular glutamate [23]. Once transported into the cell, cystine is quickly reduced to cysteine, the rate limiting substrate for *de novo* glutathione synthesis [24]. Glutathione is essential for the maintenance of cellular redox homeostasis and is often upregulated in cancer cells in response to elevated levels of ROS [25]. Subsequently, elevated expression and activity of system x_C_^-^ can increase the antioxidant capacity of cancer cells, resulting in resistance to chemotherapy [26]. In mouse models of ovarian cancer, [^18^F]FSPG was shown to predict resistance to chemotherapy through the measurement of *de novo* glutathione biosynthesis [20, 21]. Outside of cancer imaging, [^18^F]FSPG has been investigated for the imaging of multiple sclerosis [27] and cerebral ischemia [28].

The reproducible production of radiotracers in high yield and purity on automated synthesis modules is required for their clinical translation and routine preclinical use. A number of commercially available synthesis modules are currently in use for clinical and preclinical radiotracer production, including FASTlab^TM^ (GE Healthcare), TRACERlab™ FXFN (GE Healthcare), E-Z modules (Eckert & Ziegler modular lab), Explora® series (Siemens Healthcare), and the AllInOne (Trasis) [29]. Each synthesis module varies in terms of its flexibility for the creation of novel syntheses, with many optimized for [^18^F]FDG clinical production. As a result, the automation of new radiotracer syntheses on these modules is often far from trivial. Careful optimization of each reaction step and automated manipulation is key to ensuring the radiotracer is reproducibly produced with high purity and supplied in the quantities and concentrations required for preclinical or clinical imaging. Further complications can be found in research facilities with limitations on the amount of radioactivity that can be used, for example as a result of supply issues, lack of extensive radioactivity shielding (i.e. a hot cell), or limitations imposed by site licenses.

Herein, we report the automated synthesis of [^18^F]FSPG on the GE FASTlab, one of the most widely used synthesis modules for routine clinical radiotracer production. Additionally, we demonstrate how small adaptations to the production process can maximize output for preclinical studies, even when starting with a relatively low amount of radioactivity. The final radiotracer concentration is a central component in the process optimization. The synthesis method reported here reliably produced [^18^F]FSPG in good radiochemical yield (RCY) and high radiochemical purity (RCP).

## Materials and Methods

### General

Radioactivity was measured in a CRC-25R dose calibrator (Capintec Inc.). The automated radiosynthesis platform used in the study was the GE FASTlab™ (GE Healthcare), which was used in conjunction with the FASTlab Developer software (version 3.2.0.2). All experiments were performed within a lead castle made of 50 mm lead bricks. All FASTlab consumables were obtained from GE Healthcare. ‘Short tubing’ refers to Assembled Tubing Short PET, PART #: 1135059 (14 cm). ‘Long tubing’ refers to Assembled Tubing Long PET, PART #: 1135058, (42 cm). [^18^F]FSPG precursor **1** was obtained from ABX GmbH, Radeberg, Germany. QMA Carbonate Plus light SepPak (PART #: 186004051), MCX Oasis (PART #: WAT186003516), and Alumina light SepPak cartridges (PART #: WAT023561) were purchased from Waters. Hypercarb cartridges were purchased from Thermo Fisher. Unless otherwise specified, ‘water’ refers to sterile ultrapure water (18.2 MΩ-cm). PBS was prepared by dissolving one phosphate-buffered saline tablet (Product code: 1002705562, Sigma Aldrich) in 200 ml of water to produce 0.01 M phosphate buffer, 0.0027 M potassium chloride, and 0.137 M sodium chloride, with pH 7.4, at 25 °C. OPA reagent (#5061-3335) was obtained from Agilent. Analytical and semi-preparative RP-HPLC were performed on an Agilent 1200 HPLC system equipped with a 1200 Series Diode Array Detector and a GABI Star NaI(Tl) scintillation detector (energy window 400-700 keV; Raytest). Isolated RCY refers to the activity of the pure radiotracer isolated after HPLC divided by the initial activity of [^18^F]Fluoride in [^18^O]H_2_O used for the labelling. RCYs are given decay corrected. RCP refers to the proportion of the total radioactivity in the sample which is present as the desired radiotracer, as measured by radio-HPLC [30].

### Preparation of Cartridges, Reagents, and Cassette

QMA light SepPak cartridges were used as received. MCX Oasis cartridges were conditioned using PBS (10 ml) and water (20 ml) followed by air (10 ml). Alumina light SepPak cartridges were conditioned using water (10 ml) followed by air (10 ml). Hypercarb cartridges were conditioned using PBS (10 ml) and water (20 ml) followed by air (10 ml). The required consumables and reagents are described in Table 1. Reagent vials were prepared on the day of synthesis. Reagents were contained in crimp sealed glass vials and loaded onto the cassette prior to synthesis.

**Table 1.** FASTlab cassette reagent positions for the radiosynthesis of [^18^F]FSPG

Cassette position (CP)	Reagent, hardware, or consumable
1	Short tubing to ^18^O water collection vial
2	Kryptofix® carbonate solution (11 mm Vial, 850 μl)
3	Syringe 1
4	QMA light SepPak cartridge
5	Short tubing to QMA light SepPak cartridge at CP4
6	^18^F Inlet
7	Short tubing to reactor (LHS)^a^
8	Short tubing to reactor (Center)
9	Long tubing to external vial (sulfuric acid, 34 ml, 0.12 M)
10	Long tubing to external vial (PBS, 25 ml)
11	Syringe 2
12	Precursor Solution (11 mm Vial, 1.7 ml)
13	Dry acetonitrile (13 mm Vial, 1.6 ml)
14	1M Sulfuric acid (13 mm Vial, 2.0 ml)
15	Water Spike/ water bag
16	4 M Sodium hydroxide (13 mm Vial, 1.5 ml)
17	Unused
18	Alumina light SepPak cartridge (+ long tubing to Hypercarb cartridge)
19	Short tubing to MCX Oasis cartridge at CP20
20	MCX Oasis cartridge
21	Short tubing to MCX Oasis cartridge at CP22
22	MCX Oasis cartridge
23	Unused
24	Syringe 3
25	Long tubing to reactor (RHS)^b^

^a^*LHS* left hand side, ^b^*RHS* right hand side

### Integration of Hypercarb Cartridge and Switch Valve Collection

The external Hypercarb cartridge was connected to the FASTlab via a ‘long tube’ to the Alumina light SepPak cartridge at position 18. The cartridge was held securely to a clamp stand next to the FASTlab module within the lead castle. An additional ‘long tube’ was attached to the male end of the cartridge and passed through a small gap in the lead castle for connection to the switch valve for collection. The switch valve and collection vials were contained in a separate smaller shielded compartment to allow access to the product vial without exposure to the high amounts of radioactivity contained within the main lead castle.

### Optimized Automated Synthesis of [^18^F]FSPG

[^18^F]Fluoride was produced by a GE PETrace cyclotron (GE Healthcare) by 16 MeV irradiation of enriched [^18^O]H_2_O target, supplied by Alliance Medical Radiopharmacy Ltd. (London, UK) or St. Thomas’ Hospital (London, UK) in approximately 3 ml of water. [^18^F]Fluoride was provided as a full target load. Approximately 1.5 GBq of [^18^F]Fluoride was aliquoted and used without further purification or dilution. [^18^F]Fluoride was trapped on a QMA light SepPak cartridge and the ^18^O water eluted into a collection vial for recovery. Kryptofix carbonate solution [Kryptofix (8.0 mg, 21.2 μmol), potassium carbonate (1.1 mg, 8.0 μmol), acetonitrile (0.650 ml), and water (0.200 ml)] was taken up by syringe 1 and eluted through the QMA light SepPak cartridge into the reaction vessel. The [^18^F]Fluoride/Kryptofix/carbonate mixture was azeotropically dried at 120 °C under a mixture of nitrogen pressure (200 mbar) and vacuum (−1000 mbar) using anhydrous acetonitrile (3 × 0.40 ml). Next, the reactor temperature was allowed to cool to 110 °C under a flow of nitrogen and the precursor (6.0 mg, 12.2 μmol) was added to the reaction vessel as a solution in anhydrous acetonitrile (1.70 ml). Following heating at 110 °C for 10 min, the reaction temperature was lowered to 100 °C. A solution of sulfuric acid (2.0 ml, 1 M) was subsequently added, and the mixture was kept at 100 °C for 4 min. The reaction temperature was lowered to 70 °C, and a solution of sodium hydroxide (1.5 ml, 4 M) was added. After 5 min, the reaction mixture was transferred into an external vial containing a solution of sulfuric acid (34 ml, 0.12 M). Nitrogen was passed through the mixture for 20 s to encourage mixing. The crude product was extracted on to two MCX Oasis cartridges, using syringe 2 for loading in a stepwise manner. A slower loading was achieved by breaking down the syringe plunger movements into multiple programmed stop/start commands (12 × 2 second intervals). Washing the two MCX Oasis cartridges with water (28 ml) was then performed in the same fashion. Both the extraction of [^18^F]FSPG and washing steps were performed by loading onto the female end of the cartridge. Syringe 3 was used to pass the PBS eluent through the two MCX Oasis cartridges in a ‘reverse elution’, passing in the opposite direction to which the MCX Oasis cartridges were loaded and washed (12 × 5 second intervals). The product was eluted through an Alumina light SepPak cartridge and an altered Hypercarb cartridge before collection in a glass vial. The optimized FASTlab sequence is provided in the supplementary materials.

### Characterization of [^18^F]FSPG

Analytical RP-HPLC was performed on an Agilent 1200 HPLC system equipped with a 1200 Series Diode Array Detector and a Raytest GABI Star NaI(TL) scintillation detector (energy window 400–700 keV). The product was analyzed by a radio-HPLC system (Agilent 1260 series) with a variable wavelength detector and a GABI Star NaI(TL) scintillation detector—Column: Chromolith C18 (100 × 4.6 mm), Merck Millipore; solvent A: H_2_O (0.1 % TFA), solvent B: MeOH (0.1 % TFA); flow rate: 3 ml/min; UV detector: 314 nm; gradient: 10–90 % B, 0–10 min; 90 % B, 10–15 min. For UV detection, [^18^F]FSPG solution (20 μl) was added to OPA reagent (20 μl) followed by addition of PBS (80 μl). The mixture was left for 5 min prior to the HPLC analysis (Supplemental Fig. S1-S2). The molar activity was calculated by measuring the UV absorbance associated with the FSPG-OPA adduct with the following HPLC gradient conditions: 5–37 % B, 0-1 min; 37 % B, 1–21 min; 37–5 % B, 21–25 min (Supplemental Fig. S3). Molar activity (expressed in GBq/μmol) was measured using HPLC and was calculated using the equation:

$$ \mathrm{Molar}\ \mathrm{activity}=\frac{\mathrm{Activity}\ \mathrm{injected}\ \left(\mathrm{GBq}\right)}{\mathrm{Amount}\ \mathrm{injected}\ \left(\mu \mathrm{mol}\right)} $$

The amount injected was calculated using the calibration curve in Supplemental Fig. S4, assuming that the reaction of FSPG with OPA went to completion. The pH of the final [^18^F]FSPG PBS formulation was measured using Fisherbrand™ pH Indicator Paper Sticks (product number: 10642751).

### Subcutaneous Tumor Model

All animal experiments were performed in accordance with the United Kingdom Home Office Animal (scientific procedures) Act 1986. 5 × 10^6^ A549 human lung cancer cells in Dulbecco’s PBS (100 μl) were injected subcutaneously into the upper flank of female Balb/c nu/nu mice aged 6–9 weeks (Charles River Laboratories). Tumor dimensions were measured using an electronic caliper, and the volume calculated using the following equation: volume = ((*π*/6) × *h* × *w* × *l*), where *h*, *w*, and *l* represent, height, width, and length, respectively. PET imaging studies with [^18^F]FSPG took place when tumor volume reached approximately 100 mm^3^.

### PET/CT Imaging

For tumor imaging studies, mice were maintained under anesthesia with isoflurane (1.5–2 % in oxygen) at 37 °C during tail vein cannulation and throughout imaging. Dynamic PET scans were acquired on a four-bed mouse hotel [31] for 60 min following a bolus intravenous injection of ~3 MBq of radiotracer through a tail vein cannula. CT images were acquired for anatomical visualization (480 projections; helical acquisition; 55 kVp; 600 ms exposure time). Following image reconstruction (Tera-Tomo 3D; 4 iterations, 6 subsets; 0.4 mm isotropic voxel size), VivoQuant software (v 2.5, Invicro Ltd.) was used to manually remove the imaging bed from acquired CT images.

### Statistical Analysis

Statistical analysis was conducted using GraphPad Prism. All data were expressed as the mean ± one standard deviation (SD). Statistical significance was determined using a two-tailed Student’s *t*-test, with *p* < 0.05 classified as significant.

## Results

### Automated Synthesis of [^18^F]FSPG

**Fig. 1.**
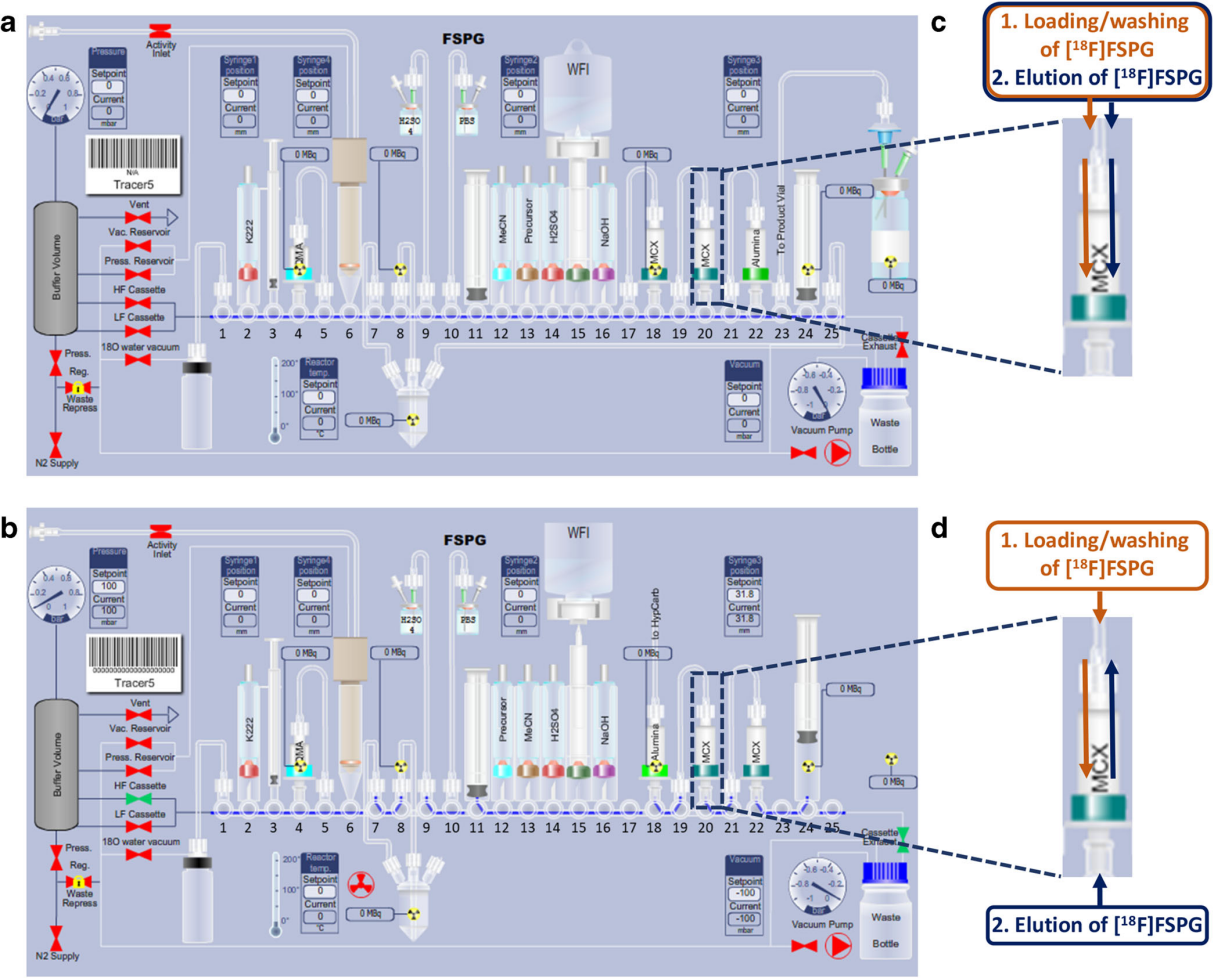
FASTlab cassette layouts for the radiosynthesis of [^18^F]FSPG. **a** Original cassette layout. **b** Optimised cassette layout to enable reverse elution. **c** ‘Normal’ loading, washing, and elution of [^18^F]FSPG. **d** ‘Normal’ loading and washing of [^18^F]FSPG, followed by a ‘reverse’ elution. Numbers refer to each individual valve or cassette position (CP). Consumables and reagents added to each position are described in Table 1. Tubing from the reactor to CP 25 is not shown for clarity. WFI, Water for injection.

The radiosynthesis of [^18^F]FSPG was conducted according to the methodology reported for the Scintomics HBIII automated synthesis module [21]. The method proceeds via a two-step synthesis (S_N_2 labeling of **1** to produce [^18^F]**2**, with subsequent deprotection yielding [^18^F]FSPG) followed by automated cartridge-based purification. Initial adaptation of the synthesis for the FASTlab was conducted using the same reaction conditions as previously reported (Table 2, Entry 1), using the cassette layout shown in Fig. 1a. Production of [^18^F]FSPG using these conditions was confirmed by radio-HPLC analysis of the crude reaction mixture, although radiochemical conversion (RCC) was relatively low (23 %). Unfortunately, elution of [^18^F]FSPG from the MCX Oasis cartridges with PBS (10 ml) was unsuccessful and a manual elution of the radiotracer was required providing [^18^F]FSPG with low RCP (87 %). Furthermore, [^18^F]FSPG was observed in the waste collection bottle, suggesting issues with either the loading of the radiotracer on to the MCX Oasis cartridges or the subsequent washing steps.

**Table 2.**
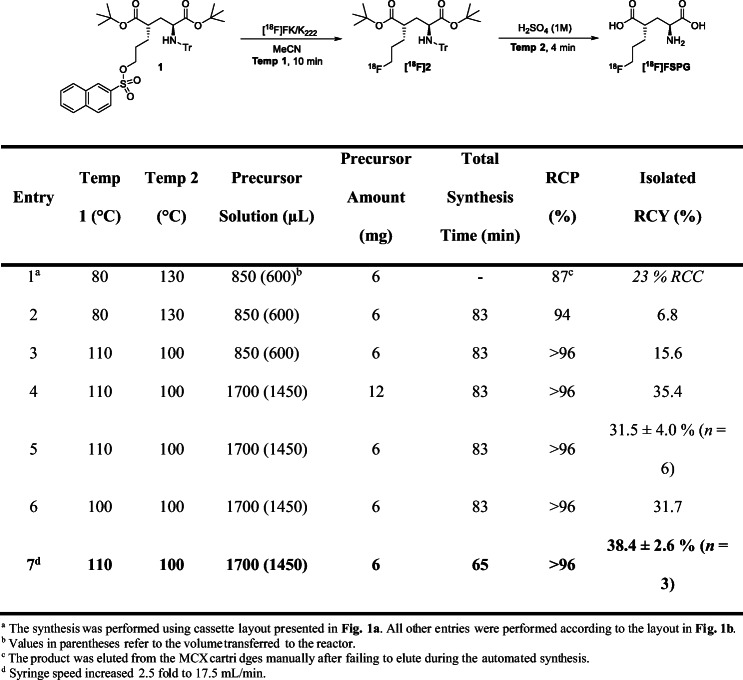
Optimization of the reaction conditions for the synthesis of [^18^F]FSPG

### Reverse Elution of [^18^F]FSPG

Changes were made to the automated sequence and cassette layout in order to ensure an improved elution of [^18^F]FSPG during the automated process (Fig. 1b). Rather than eluting the [^18^F]FSPG through the two MCX Oasis cartridges, as illustrated in Fig. 1c, the PBS was passed backwards through the MCX cartridges, eluting the radiotracer back off of the top of the cartridge it was loaded on to in a ‘reverse elution’ (Fig. 1d). Furthermore, to improve the trapping efficiency of the solid phase extraction, the loading speed of the crude product from the external quenching vial and all subsequent washing and elution steps were reduced from 76 ml/min (the pre-set speed of the syringe FASTlab syringe drives) to 7 ml/min. As the syringe driver could not be directly controlled, this slower elution was achieved by breaking down the syringe movements into multiple programmed stop/start commands (12 × 5 second intervals were used for syringes 2 and 3, with a total volume of 7 ml). These adjustments to the process resulted in a fully automated synthesis and isolation of [^18^F]FSPG with an improved RCP of 94 %, although the isolated RCY was low, at 6.8 % (Table 2, Entry 2).

### Optimization of RCY and RCP

Optimization was conducted to improve the RCY using the newly established automated protocol: increasing the temperature of the labelling reaction to 110 °C improved the RCY to 15.6 % whilst reduction of the deprotection temperature was tolerated and gave an improved RCP of 96 % (Table 2, Entry 3). We found that an increase in the volume of the radiolabeling reaction mixture was key to improving the RCY. Initially, the amount of precursor was doubled in order to maintain precursor concentration (Table 2, Entry 4). These conditions resulted in a much-improved RCY of 35.4 %. It was found, however, that reduction to the original precursor amount could be tolerated when the reaction volume was doubled, resulting in a RCY of 31.5 % ± 4.0 % (Table 2, Entry 5; *n* = 6). Decreasing the temperature of the labelling reaction to 100 °C did not affect RCY, and therefore, the reaction temperature was maintained at 110 °C for further optimization (Table 2, Entry 6). Finally, further adjustments were made to the automation to reduce the synthesis time. The water wash was reduced from 60 ml to 28 ml, whilst increasing the elution and washing speed from 7.5 ml/min to 17.5 ml/min. Including both of these adjustments reduced the synthesis time from 83 minutes to 65 minutes and did not result in any change to RCP or formulation pH, which was between 5 and 6. These changes also resulted in an improved RCY of 38.4 ± 2.6 % (Table 2, Entry 7), corresponding to a non-decay corrected RCY of approximately 25.5 % and the production of 382.5 MBq of [^18^F]FSPG at the end of synthesis (EOS) from 1.5 GBq starting activity. The conditions in Table 2 (Entry 7) provided the optimal balance between the RCY, total synthesis time, and the relative expense of the precursor.

### Improving Radioactive Concentration

As well as optimizing RCY and RCP, our aim was also to increase the radiotracer concentration to maximize the number of small animal scans that could be performed with each [^18^F]FSPG production. [^18^F]FSPG was produced in our laboratory on a synthesis module that was housed in a lead castle not a hot cell. This limited the amount of starting activity to 1.5 GBq, removing the possibility of improving the radiotracer concentration by starting the synthesis with a higher amount of radioactivity. More than 20 ml of PBS buffer was required to elute the radiotracer from the MCX Oasis cartridges, and this large volume resulted in a radiotracer concentration of <10 MBq/ml at EOS. In order to avoid a manual reformulation, we sought to improve the radiotracer concentration by optimizing the isolation and collection of [^18^F]FSPG.

**Fig. 2.**
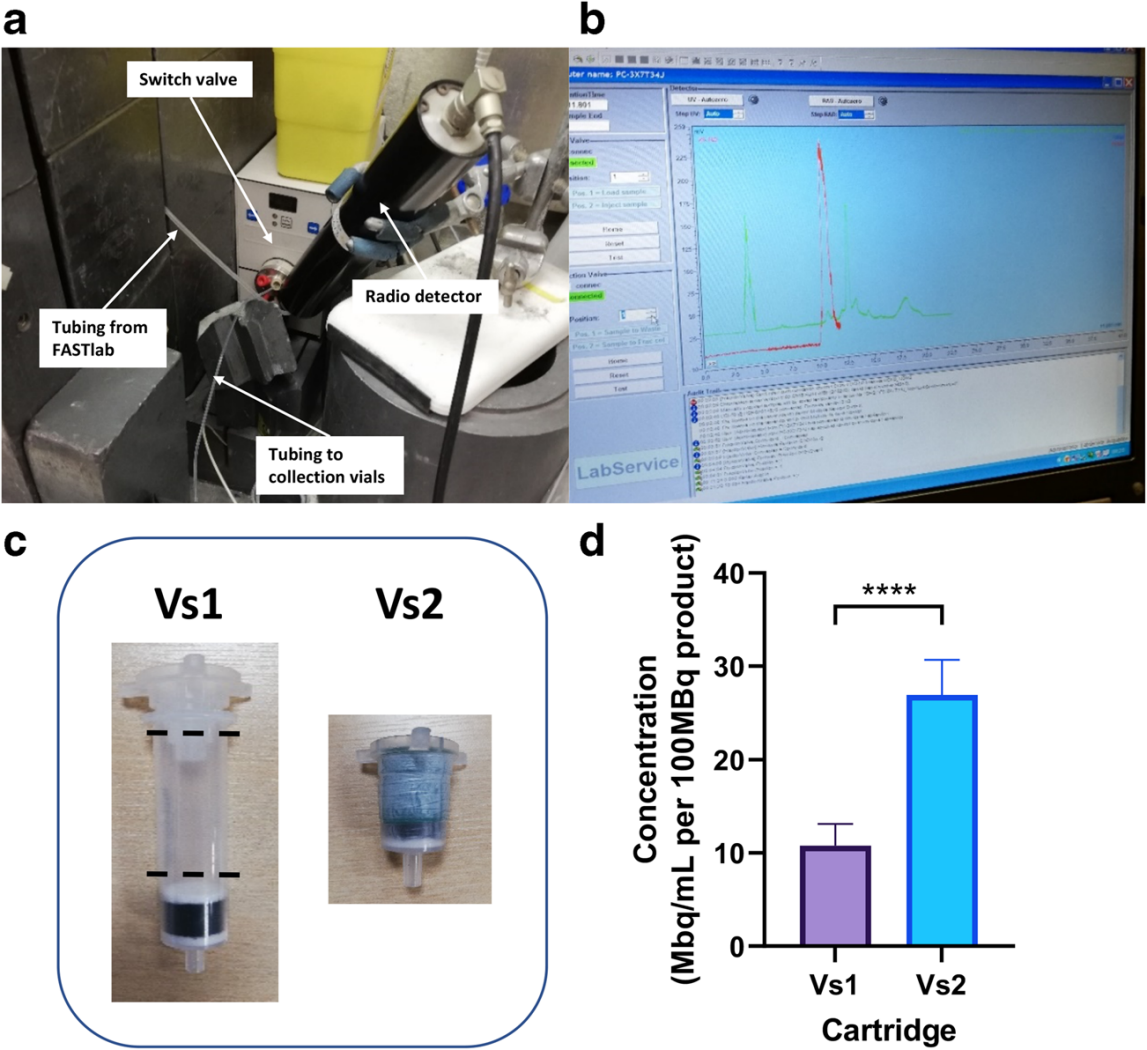
Optimization of radiotracer concentration. **a** Set-up of switch valve and radio detector outside the ‘lead castle’ enclosing the FASTlab to facilitate remote collection at the point of highest radioactive concentration. **b** Real-time output of radioactivity detector during the elution process (radioactivity trace is in red). **c** Hypercarb cartridges Vs1 and Vs2. Dotted lines indicate the dead volume removed from Vs1 to produce Vs2. **d** Impact of Hypercarb cartridge Vs1 and Vs2 on radiotracer concentration. Concentration was normalized to total [^18^F]FSPG radioactivity (MBq/ml per 100 MBq of product) in order to account for any differences in starting activity or RCY. Error bars are SD. ****P < 0.0001.

To lower the eluent volume, the product outlet was connected to a switch valve, external to the main lead castle, where radioactivity could be measured with an external detector (Fig. 2a). This allowed for remote collection of the most concentrated fraction of eluent (Fig. 2b) and improved the radioactive concentration more than 3-fold to ~30 MBq/ml (*n* = 7). A noticeable limitation in the cartridge-based purification was the large dead volume of the commercially available Thermo Fisher Hypercarb cartridge (Vs1; Fig. 2c). [^18^F]FSPG was passed through the Hypercarb cartridge in the final step of the cartridge purification. The Hypercarb cartridge is not designed for automated synthesis and consequently requires two adaptors to link the cartridge to the FASTlab tubing. The large dead volume found between the adaptors and the solid phase component of the cartridge led to accumulation of the eluent and resulting dilution of the most concentrated fraction. In order to overcome this problem, the dead volume was removed to produce an altered Hypercarb cartridge (Vs2; Fig. 2c), more suitable for automated purification. For Vs2 assembly details, see Supplemental Fig. S5. To compare Vs1 to Vs2, resulting radiotracer concentrations (MBq/ml) were normalized to 100 MBq of [^18^F]FSPG produced in order to account for any differences in starting activity or RCY. The use of Vs2 resulted in a considerable increase in radiotracer concentration, more than doubling that produced using Vs1 from 10.8 ± 2.32 MBq/ml to 26.9 ± 3.75 MBq/ml per 100 MBq of product, respectively (*n* = 7 and 12 respectively; *P* ≤ 0.0001; Fig. 2d). With Vs2, the non-normalized radiotracer concentration achieved with the optimized radiosynthesis conditions was 102 ± 14.3 MBq/ml (*n* = 3) when starting with 1.5 GBq [^18^F]Fluoride in a final volume of approximately 3.5 ml.

### Characterization of [^18^F]FSPG

Radio-HPLC analysis of the produced [^18^F]FSPG showed >96 % RCP. Further analysis after 6h confirmed that the product was stable (>96 % RCP; Supplemental Fig. S6). The molar activity of the [^18^F]FSPG produced using the optimized synthesis was 11.1 ± 7.7 GBq/μmol (*n* = 4).

### The Impact of Radiotracer Concentration on Preclinical Imaging Studies

**Fig. 3.**
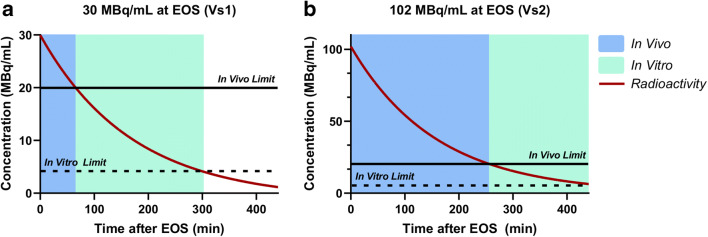
Modeling [^18^F]FSPG concentration and preclinical utility. **a** [^18^F]FSPG concentration and utility over time when using Hypercarb Vs1. **b** [^18^F]FSPG concentration and utility over time when using Hypercarb Vs2*.*

To understand the impact of radiotracer concentration on our imaging studies, the maximum preclinical imaging time was calculated from the concentrations of [^18^F]FSPG achieved at EOS during each stage in our method optimization (see Table 3). Calculations were based on an i.v. injection of 3.0 MBq of [^18^F]FSPG in a maximum volume of 150 μl. Despite reproducible production of [^18^F]FSPG in good RCY and high RCP, small animal imaging could not be conducted after optimization of the reaction conditions alone (Table 3, Entry 1). Implementation of an external remote collection of the radiotracer achieved radiotracer concentrations high enough to perform *in vivo* imaging studies (30 MBq/ml; Table 3, Entry 2). Modeling the decay of radiotracer concentration, however, revealed that imaging could only be performed up to 65 minutes after EOS, limiting 1 h dynamic imaging acquisitions to 1 or 2 mice (Fig. 3a). Further improvements to the radiotracer concentration by altering the Hypercarb cartridge (76 MBq/ml; Table 3, Entry 3) substantially increased preclinical output. In combination with improvements to the reaction RCY, the radiotracer concentration was increased to 102 MBq/ml. Imaging could now be performed up to 255 min after EOS (Fig. 3b), facilitating imaging of up to five mice using a standard small animal imaging bed or 20 mice using the 4-bed mouse ‘hotel’ (Table 3, Entry 4).

**Table 3.** Changes made to [^18^F]FSPG isolation and their effect on preclinical imaging studies

Entry	Improvements implemented	Concentration at EOS (MBq/ml)^a^	Preclinical imaging time (min)	Number of mice imaged ^b^
1	-	<10^c^	Cell work only	0
2	Remote Collection	30^c^	65	1-2 (4–8)
3	Hypercarb Vs2	76^c^	210	4 (16)
4	Syringe speed	102^d^	255	5 (20)

^a^Concentration refers to the average concentration of [^18^F]FSPG produced when using a starting activity of 1.5 GBq

^b^The number of mice that can be scanned for a standard 1h dynamic acquisition. Parentheses refer to the number of mice that can be scanned for a standard 1 h acquisition using a 4-bed mouse ‘hotel’ developed in our lab [31]

^c^Concentrations obtained using conditions reported in Table 2, Entry 5

^d^Concentrations obtained using conditions for the fully optimized process reported in Table 2, Entry 7

**Fig. 4.**
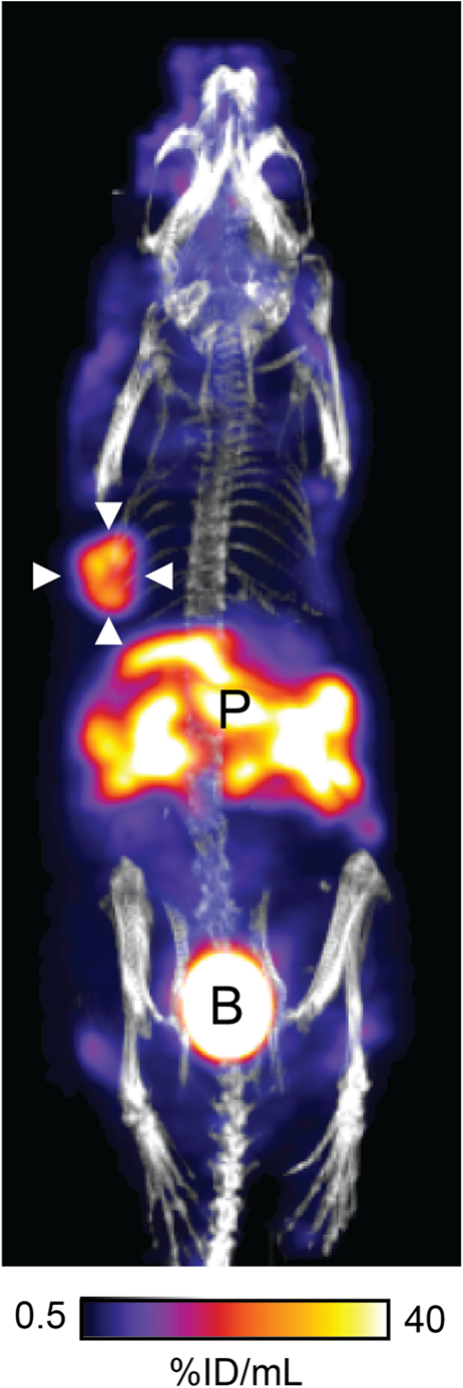
*In vivo* [^18^F]FSPG PET/CT imaging of a mouse bearing a subcutaneous A549 tumor. Maximum intensity projection 40–60 min after [^18^F]FSPG injection following the manual removal of the bed. White arrowheads indicate the tumor margins. P, pancreas; B, bladder.

The [^18^F]FSPG produced using our optimized synthesis protocol was used to image tumors in a subcutaneous A549 non-small cell lung cancer model. Representative static [^18^F]FSPG PET/CT maximum intensity projection of 40–60 min summed activity (Fig. 4) revealed typical [^18^F]FSPG biodistribution, characterized by high uptake in the tumor and pancreas, with radiotracer excreted via the urinary system [20, 21]. No uptake was observed in the bone, indicating the absence of free [^18^F]Fluoride.

## Discussion

The altered expression and activity of system x_C_^-^ in cancer have enabled the use of [^18^F]FSPG for tumor detection in various cancer types [8, 9, 13]. To facilitate the use of [^18^F]FSPG in preclinical and clinical studies, reliable automated synthesis of the radiotracer is necessary. Our aim was to develop an automated procedure for the synthesis and purification of [^18^F]FSPG on a single synthesis module that would yield [^18^F]FSPG in sufficient radiotracer concentration for preclinical imaging, even when starting with low amounts of radioactivity. To facilitate rapid translation of this methodology and to distribute to multiple sites, we selected the GE FASTlab for automated radiosynthesis. As one of the most widely used synthesis modules for clinical production, the FASTlab is well-equipped to enable rapid cartridge-based purifications suitable for GMP synthesis and additionally has been used for the automated synthesis of a variety of ^18^F-labeled radiotracers [32–35].

[^18^F]FSPG has been synthesized previously using the Eckert and Ziegler modular lab [7–9, 12, 19], the GE TRACERlab [11, 15], and the Scintomics HBIII platform [20, 21]. We started by adapting the automated synthesis procedure using the previously published Scintomics HBIII platform methodology [21]. Whilst [^18^F]FSPG could be synthesized using this initial set-up, problems with isolation of the produced [^18^F]FSPG prompted us to redesign the cassette layout. The second cassette layout (Fig. 1b) allowed for the ‘reverse elution’ of the radiotracer from the MCX cartridges on which it was trapped and washed. This meant that a smaller volume of eluent was required for elution and a more concentrated fraction was collected. Although standard elution in large volumes of eluent may be sufficient for clinical studies using higher starting activities, the production of a highly concentrated product may facilitate transportation to multiple sites distant from the site of synthesis. Under these circumstances, however, care should be taken to prevent any potential radiolysis that might occur.

Optimization of the reaction conditions quickly increased both RCY and RCP (Table 2). After adjustment of the reaction temperatures and reaction mixture volumes, [^18^F]FSPG was obtained in 31 ± 4 % (*n* = 6) RCY and >96 % RCP. Subsequently, optimization of the automated process focused on decreasing the synthesis time. Careful adjustment of the elution flow rate and reduction in the volume of water used to wash the cartridge-bound radiotracer reduced the synthesis time by 18 min without compromising on the RCP (>96 %) and led to an improved RCY of 38.4 ± 2.6 % (*n* = 3).

In order to perform *in vivo* imaging with the produced [^18^F]FSPG, a minimum radioactive concentration of approximately 20 MBq/ml was required. Due to the large volume of eluent used to elute and isolate the radiotracer from the solid phase extraction (SPE) cartridges, initial radiotracer concentrations were far lower than 20 MBq/ml. Adjustments were made to the isolation procedure in order to overcome these issues. Firstly, an external detector and switch valve were employed to perform remote collection of the most concentrated fraction of the elution. This facilitated the isolation of [^18^F]FSPG at concentrations suitable for preclinical imaging, although the number of animals that could be imaged at this concentration (2 mice) was limited by the short half-life of the ^18^F radionuclide. Further improvements to radiotracer concentration were achieved by altering the Hypercarb cartridge used in the solid phase extraction purification process (Fig. 2c). By removing the large dead volume of the cartridge, dilution effects were minimized, leading to a substantial increase in radiotracer concentration (~102 MBq/ml). A large number of mice (~20) could be scanned with this radiotracer concentration when using a 4-bed mouse ‘hotel’ (Fig. 3b), and transportation of the radiotracer to other research institutes across London was possible.

The final optimized sequence provided [^18^F]FSPG in 38.4 ± 2.6 % RCY in approximately 1 h, producing ~383 MBq of [^18^F]FSPG from 1.5 GBq starting activity without the use of a hot cell. Whilst our work has focused on optimizing this process for preclinical studies, we anticipate only minor adaptation of the sequence being required for clinical translation. To our knowledge, this method is the first cassette-based synthesis and purification of [^18^F]FSPG. Omission of the external switch valve, designed to improve radiotracer concentration only when low starting activities are required, will allow for ‘plug and play’ operation that is desirable for GMP production. For clinical translation, a different formulation that does not contain PBS may also be required. Despite these considerations, [^18^F]FSPG was produced in amounts greater than that required for a patient dose (300 MBq). This has particular relevance to a ‘dose on demand’ cyclotron, where the amount of produced [^18^F]Fluoride is in the range of 1-2 GBq [36]. Additionally, the RCP of [^18^F]FSPG was markedly higher than those used in previous clinical trials (>90 %) [7, 9, 15] which may be due to the absence of radiolysis. Further studies are required to understand the interplay between [^18^F]FSPG radiochemical concentration and RCP, but this work provides tantalizing evidence that multiple patient doses of [^18^F]FSPG could be prepared when high starting activities are used. Here, we have demonstrated how practical changes to an automated procedure can result in significant improvements in research output whilst maintaining the ease of cassette-based radiochemistry. Specifically, we have demonstrated how barriers to PET imaging research can be overcome when low levels of starting activity are available.

## Conclusion

The synthesis of [^18^F]FSPG was automated and optimized on the GE FASTlab synthesis module. It was found that the radiotracer concentration was a limiting factor for preclinical imaging studies performed in our setting. These issues were overcome by applying small alterations to the automated protocol. Implementation of a reverse elution, an improved remote collection, and alteration of the commercially available Hypercarb cartridge used for the solid phase extraction purification of the radiotracer resulted in a considerable improvement in radiotracer concentration and RCY. This optimized protocol reliably produced [^18^F]FSPG in high RCY (38.4 ± 2.6 %) and RCP (>96 %) with a molar activity of 11.1 ± 7.7 GBq/μmol. Furthermore, the high radiotracer concentrations achieved allowed for large-scale *in vivo* studies and transportation to other sites despite low levels of starting radioactivity. The optimized protocol will increase accessibility to [^18^F]FSPG and is well-placed for clinical translation.

## Supplementary Information

ESM 1 (DOCX 566 kb)

ESM 2 (ZIP 19.9 KB)
