# Supplementary material for: Robust and Facile Automated Radiosynthesis of [18F]FSPG on the GE FASTlab
Source: Mol Imaging Biol. 2021 May 20;23(6):854–64. doi: 10.1007/s11307-021-01609-w (PMC8578107; doi:10.1007/s11307-021-01609-w)
Supplement: Supplementary file 1 — (DOCX 566 kb) [file 11307_2021_1609_MOESM1_ESM.docx]

**Electronic Supplementary Information**

**Robust and facile automated radiosynthesis of [^18^F]FSPG on the GE FASTlab**

Richard Edwards, Hannah E. Greenwood, Graeme McRobbie, Imtiaz Khan & Timothy H. Witney

**Synthesis sequence and cassette layout**

The sequence and cassette layout are available to download and are compatible with version 2 and above of the FASTlab software.

**Supplementary Figures**


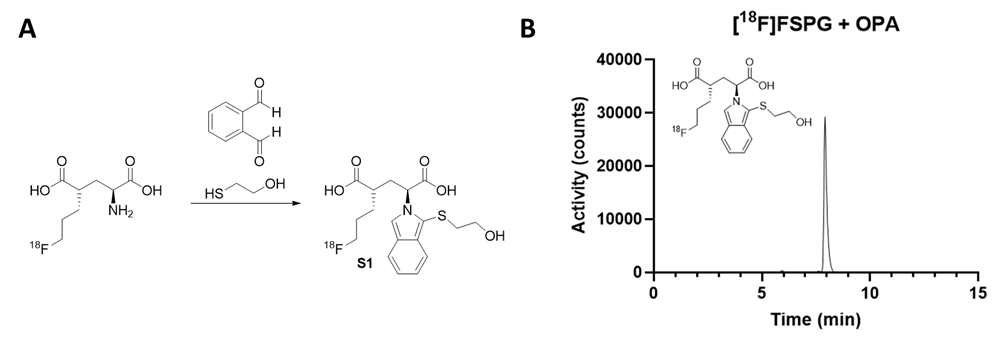


**Supplementary Fig. S1. Reaction of [^18^F]FSPG with OPA reagent.** **A)** Reaction scheme. **B)** Example chromatogram of [^18^F]FSPG after reaction with OPA. Column: Chromolith C18 (100 x 4.6 mm); solvent A: H_2_O (0.1 % TFA), solvent B: MeOH (0.1 % TFA); flow rate: 3 mL/min; UV detector: 314 nm; gradient: 10-90 % B, 0-10 min; 90% B, 10-15 min.


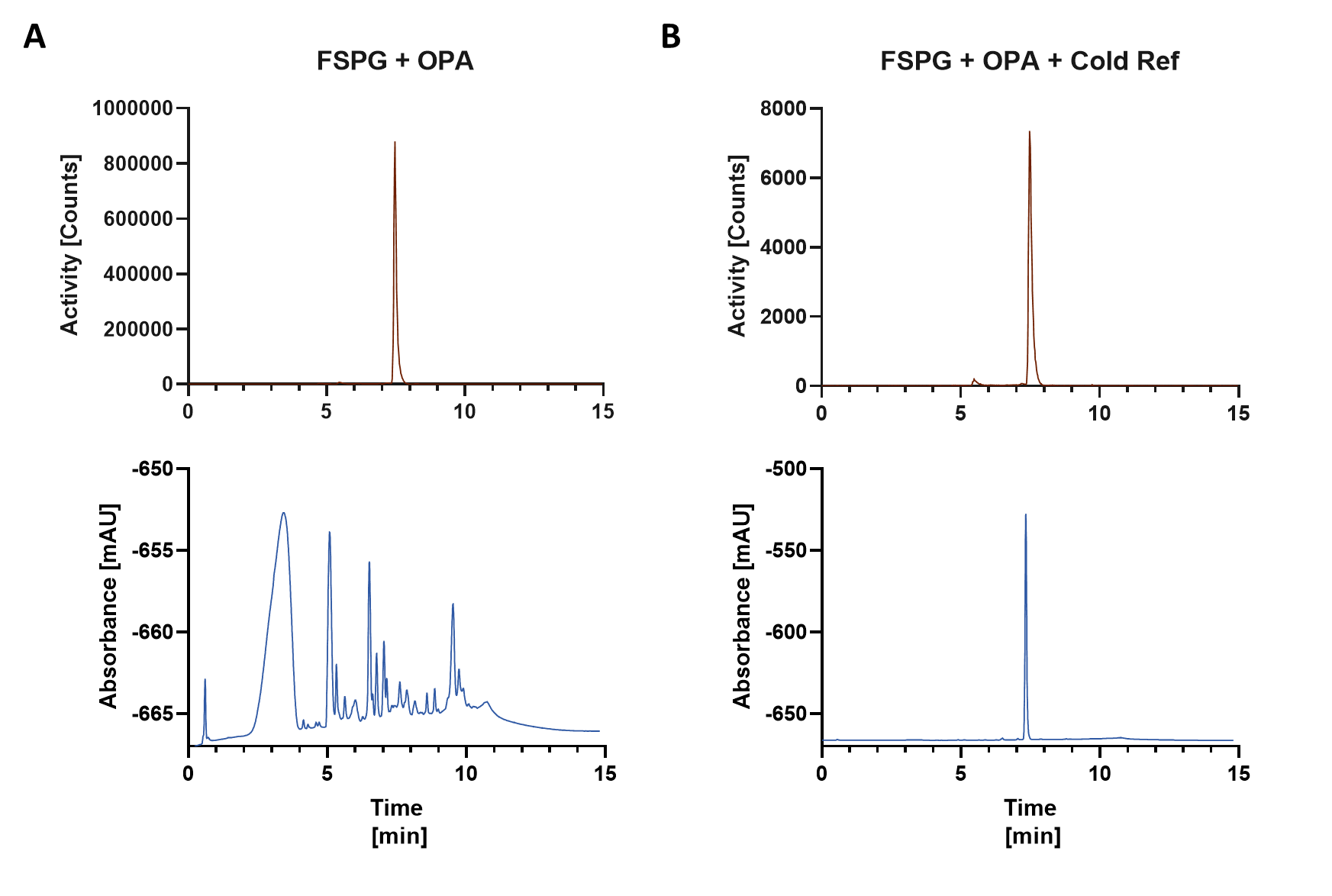


**Supplementary Fig. S2. Confirmation of product Identity. A)** Example activity and UV chromatograms of [^18^F]FSPG after reaction with OPA. **B)** Co-elution of ‘cold’ FSPG-OPA adduct **S1** after addition of cold FSPG standard to the sample mixture. Column: Chromolith C18 (100 x 4.6 mm); solvent A: H_2_O (0.1 % TFA), solvent B: MeOH (0.1 % TFA); flow rate: 3 mL/min; UV detector: 314 nm; gradient: 10-90 % B, 0-10 min; 90% B, 10-15 min.


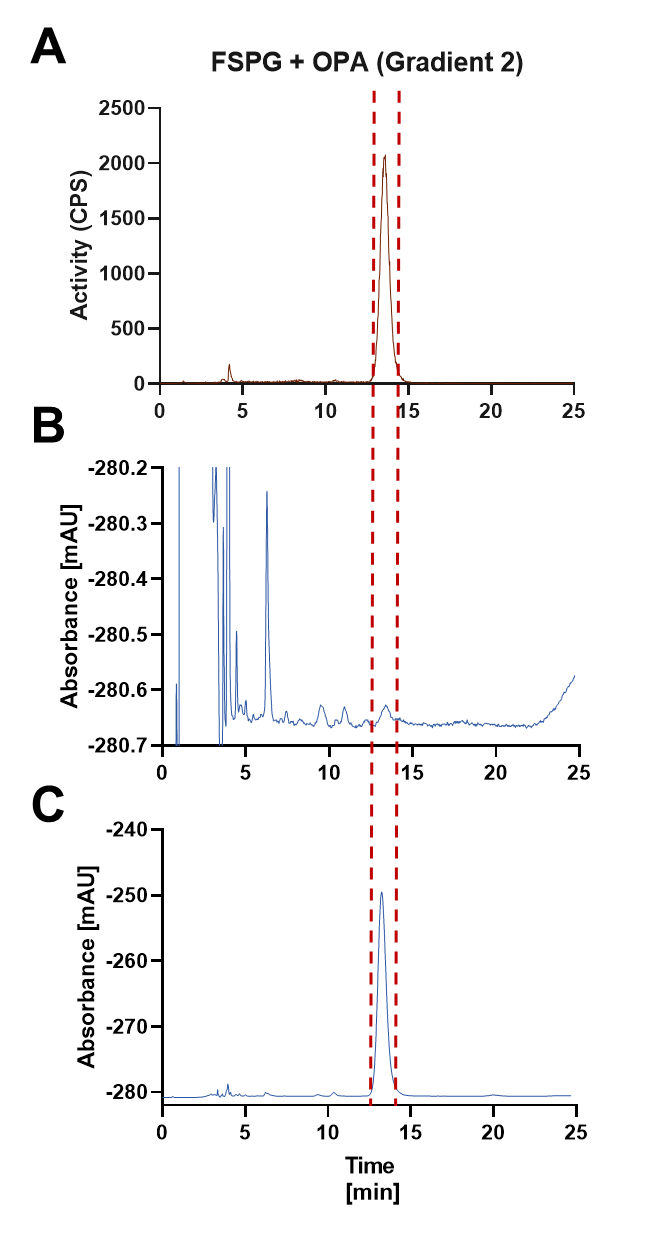


**Supplementary Fig. S3. Measurement of molar activity. A)** Example chromatogram of [^18^F]FSPG after reaction with OPA. **B)** Example chromatogram of the UV trace used to measure the molar activity of [^18^F]FSPG. **C)** Example UV trace chromatogram after addition of ‘cold’ FSPG to the OPA/[^18^F]FSPG reaction mixture. Column: Chromolith C18 (100 x 4.6 mm); solvent A: H_2_O (0.1 % TFA), solvent B: MeOH (0.1 % TFA); flow rate: 3 mL/min; UV detector: 314 nm; gradient: 5-37 % B, 0-1 min; 37% B, 1-21 min; 37-5 % B, 21-25 min.

**Supplementary Fig. S4. HPLC UV calibration curve for FSPG-OPA** (**S1**).


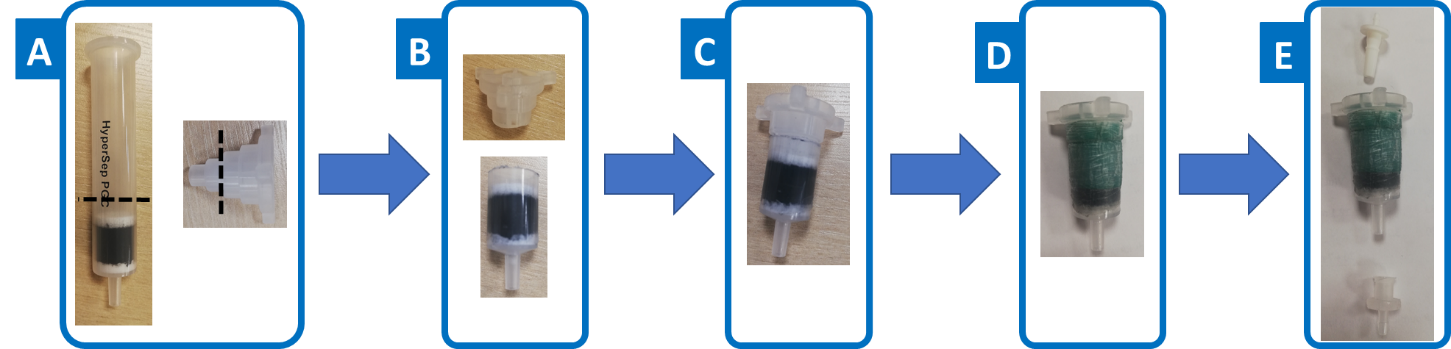


**Supplementary Fig. S5. Adaption of the Hypercarb cartridge to maximize radiochemical concentration. A)** Hypercarb cartridge (product number: 60106-402) and Adaptor Cap (for 1.3 and 6 mL SPE Tubes, product number: AH0-7191). Dashed lines show where the items were altered using a hacksaw. **B)** and **C)** Altered components of the new Hypercarb cartridge sit flush when assembled avoiding unnecessary dead volume. **D)** The cartridge components were secured with adhesive tape. **E)** Use of a Male Slip Luer to Hose Barb Adapter (product number: WZ-45504-45) and Barbed Female Luer Adapter (product number: UY-02024-06) allowed for connection to the ‘long tube’ from the FASTlab cassette and a second ‘long tube’ connected to the switch valve.

**Supplementary Fig. S6. Analysis of [^18^F]FSPG stock at 6 h post synthesis.** Chromatogram of [^18^F]FSPG after reaction with OPA 6 hours after radiotracer preparation. Initial radiotracer concentration: 95 MBq/mL. Column: Chromolith C18 (100 x 4.6 mm); solvent A: H_2_O (0.1 % TFA), solvent B: MeOH (0.1 % TFA); flow rate: 3 mL/min; UV detector: 314 nm; gradient: 10-90 % B, 0-10 min; 90% B, 10-15 min.
